# Philadelphia Academy of Surgery

**Published:** 1895-07

**Authors:** 


### Society Reports.

### PHILADELPHIA ACADEMY OF SURGERY.

Stated Meeting, May 6, 1895.

The President, Dr. Thomas G. Morton, in the chair.

Dr. H. R. Wharton exhibited a case of incised

### FRACTURE OF THE PATELLA IN WHICH WIRE WAS EMPLOYED.

Case I.—The patient was a man about 30 years of age, who was brought into the Presbyterian Hospital, June 9, 1894, with an incised wound of the knee. He said that while running, a cleaver was thrown at him, which divided the patella almost transversely and laid open the joint. I saw him a few hours after the accident. I washed out the joint and put one or two heavy silver wire sutures through the fragments of the patella and sutured the capsule with a few stitches of catgut. He was kept five weeks in bed and returned to his work in thirteen weeks. He has regained perfect motion in the joint and can flex and extend the leg and has no trouble in walking.

### ALSO A PARTIAL AMPUTATION OF THE FOOT.

Case II.—This case was injured by being run over by an engine. He was brought to the Hospital in September, 1892, and I saw him a very short time afterwards. I found that he had a complete crush of the anterior part of one foot. I did a Syme amputation, and have not seen the man since the operation until this evening. He wears an artificial apparatus and walks very well and is employed on the railroad as a switch-tender. He has a very good bearing stump. There is a good elastic pad under the bone. The apparatus is simply a shoe with a brace, with which he gets along very well. He is actively employed and has to run to throw switches. In walking the weight of the body comes directly upon the heel.

ALSO A SIMULTANEOUS AMPUTATION OF THE LEG AND PARTIAL OF FOOT.

CASE III.—This case is a man who was thrown under a car and both wheels passed over his left foot and crushed the right leg and made a compound fracture of the left thigh, requiring removal of part of the shaft of the bone. I made an amputation of the leg at the upper third and a partial amputation of the other foot. In the foot I first thought I would do a Pirogoff, but decided to leave a portion of the astragalus in place and to saw through the os calcis and bring its sawn surface in contact with the astragalus. It is seen that he has some motion in the ankle joint. He wears a peg leg on one side and a short shoe on the other foot (the left). All the force of walking and weight of his body comes on the pad on the sole of his foot.

It seems as if there is an advantage in having some ankle joint motion. If he should wear a properly made shoe, it might be possible for him to have even better motion than he has. He can now walk between five and six miles every day without trouble.

I am glad to show these cases of partial amputation of the foot, because there is a tendency at the present day to cry down this form of amputation. Many surgeons think that if part of the foot is destroyed it is better to make an amputation at what is called the point of election in the upper third of the leg. I have done many cases of this kind, but I have not seen a man who came into the hospital with a crush of the foot who would not prefer to have a partial amputation of the foot to an amputation of the leg above. I have seen several cases after Chopart's operation in which the results were very good, and the patients could walk or run without trouble.

DISCUSSION.

THE PRESIDENT: It would very much add to the comfort of this man if he would have a rubber cap on the end of his peg leg. Such an addition is commonly very satisfactory.

DR. JOHN ASHHURST: I asked the question if this patient could run. The members will remember that in a paper written by the late Dr. Addinell Hewson, he claimed as an advantage of Pirogoff's amputation that the patient was able to run as well as to walk. The same claim has been made for amputation by Syme's method, and in such a case as the present, which may be called one of Hancock's am putation, the ankle joint being preserved, the running power should also be maintained. I observe that this patient can run, though not very gracefully.

DR. RICHARD H. HARTE presented two patients.

OPERATION FOR THE RADICAL CURE OF HERNIA.

The patients were aged 16 and 50 years, respectively. Both were hospital cases upon whom I did a modification of the Italian operation. The operation was not exactly a Halsted. In the boy there was a scrotal hernia upon both sides. I did the operation first upon the left side and repeated it on the right in the course of a few days. In the boy the hernia had existed since his birth. I used kangaroo tendon in both cases and found great difficulty in getting it perfectly sterilized. It is unfit for use as it comes from the shops in oil. I took it out of the oil and put it in corrosive ether 1-2000. The last case I had became infected from the sutures.

In these cases I did not reduce the size of the cord as it did not need it. Where there is a redundancy of tissue, I do reduce it, but I did not find it necessary in either of these cases.

DR. WILLIAM J. TAYLOR: With regard to the kangaroo tendon, Dr. Harte speaks of that which is kept in oil. Now, the German authorities have found that where a substance is kept in oil it is much more difficult to disinfect and much more liable to infect than where no oil is used. I have used for some time the kangaroo tendon from the makers who prepare it in alcohol under high pressure. It is the same that is used by Dr. Coley of New York. It can be used directly from the solution in which it comes from the manufacturers and I have seen no bad results from it; but I have seen bad results follow the use of the preparation of kangaroo tendon according to the method recommended by Dr. Marcy, of Boston.

DR. R. H. HARTE: I imagine that Dr. Taylor misunderstood me with regard to the use of the tendon. I took it out of the little box in which it came and treated it as stated.

